# Agentic Artificial Intelligence in Cosmetic Dermatology

**DOI:** 10.1111/jocd.71033

**Published:** 2026-07-02

**Authors:** Mohamad Goldust

**Affiliations:** ^1^ Department of Dermatology University of Illinois at Chicago, College of Medicine Chicago Illinois USA

**Keywords:** agentic artificial intelligence, cosmetic dermatology, esthetic dermatology

Dear Editor,

Artificial intelligence (AI) is rapidly transforming cosmetic dermatology and offers applications that range from facial analysis and treatment planning to outcome prediction and patient engagement. While current AI systems primarily serve as assistive tools, a paradigm shift is emerging with the development of agentic AI, systems capable of autonomous decision‐making, goal‐directed behavior, and continuous learning. This evolution could redefine the delivery of esthetic care by enabling more adaptive, personalized, and efficient interventions [1].

Agentic AI refers to systems that operate with a degree of autonomy, integrating perception, reasoning, and action to achieve predefined objectives. Unlike traditional rule‐based or predictive AI models, agentic systems can repeatedly refine their strategies based on real‐time feedback. In cosmetic dermatology, this capability has profound implications. For example, an agentic AI platform could longitudinally analyze a patient's facial features, skin quality, and aging trajectory while considering lifestyle, environmental factors, and previous treatment responses to dynamically optimize esthetic plans [2].

Currently available AI technologies in cosmetic dermatology are largely limited to assistive and predictive functions, such as facial imaging analysis, automated skin assessment, and treatment simulation platforms. In contrast, agentic AI represents a future‐oriented concept in which systems may autonomously adapt treatment strategies, integrate longitudinal patient data, and provide dynamic decision support with minimal human prompting.

One of the most promising applications of agentic AI lies in personalized treatment. Currently, esthetic practices often rely on occasional assessments and clinician experience to guide interventions such as neuromodulators, fillers, laser therapies, and energy‐based devices. Agentic AI could continuously integrate multimodal data, including high‐resolution imaging, biometric parameters, and patient‐reported outcomes to recommend precise timing, dosing, and combinations of treatments. This approach could minimize variability in outcomes and enhance the consistency of esthetic results.

Moreover, agentic AI may enhance procedural precision and safety. By interfacing with augmented reality (AR) and robotic‐assisted delivery systems, AI agents could assist in identifying optimal injection sites, vascular anatomy, and risk areas in real time. This is particularly valuable in complex or high‐risk regions, like the glabella or nasolabial folds, where complications such as vascular occlusion can occur. Continuous intra‐procedural feedback loops would allow the system to adjust recommendations based on subtle changes in tissue response [3].

Another critical aspect is longitudinal outcome optimization. Esthetic results are inherently dynamic, influenced by biological aging, metabolic factors, and environmental exposures. Agentic AI systems could serve as “digital esthetic companions,” tracking patients over time and proactively suggesting maintenance interventions before visible deterioration occurs. This shift from reactive to anticipatory care matches broader trends in precision medicine and preventive dermatology.

Despite these advantages, several challenges must be addressed before widespread implementation. Data privacy and security are important, especially given the sensitive nature of facial imaging and personal health data. Regulatory frameworks need to evolve to define accountability in situations where autonomous systems contribute to clinical decisions. Additionally, algorithmic bias remains a concern, particularly in esthetic dermatology, where standards of beauty can be culturally and ethnically diverse. Ensuring representative training datasets will be essential to avoid inequitable outcomes [4]. Importantly, clinician oversight should remain central to all AI‐assisted decision‐making processes to ensure patient safety, ethical implementation, and appropriate interpretation of AI‐generated recommendations.

Importantly, the integration of agentic AI should not diminish the role of dermatologists; rather, it should augment their clinical expertise. Esthetic medicine is not only a technical discipline; it also requires judgment, artistic sensibility, and patient‐centered communication. The optimal model will likely be synergistic, where AI enhances precision and efficiency while clinicians retain oversight and contextual decision‐making [5].

In conclusion, agentic AI represents a transformative advancement in cosmetic dermatology, with the potential to shift the field toward autonomous, adaptive, and highly personalized care. As these systems evolve, careful attention to ethical, regulatory, and clinical considerations will be necessary to ensure that innovation translates into safe and equitable patient outcomes.

## Funding

The author has nothing to report.

## Conflicts of Interest

The author declares no conflicts of interest.

## Data Availability

Data sharing not applicable to this article as no datasets were generated or analysed during the current study.
